# Resonant Transducers Consisting of Graphene Ribbons with Attached Proof Masses for NEMS Sensors

**DOI:** 10.1021/acsanm.3c03642

**Published:** 2023-12-01

**Authors:** Xuge Fan, Daniel Moreno-Garcia, Jie Ding, Kristinn B. Gylfason, Luis Guillermo Villanueva, Frank Niklaus

**Affiliations:** † Advanced Research Institute of Multidisciplinary Sciences, Beijing Institute of Technology, Beijing 100081, China; ‡ Division of Micro and Nanosystems, School of Electrical Engineering and Computer Science, KTH Royal Institute of Technology, SE-10044 Stockholm, Sweden; § Advanced NEMS Group, École Polytechnique Fédérale de Lausanne (EPFL), 1015 Lausanne, Switzerland; ∥ School of Integrated Circuits and Electronics, Beijing Institute of Technology, Beijing 100081, China

**Keywords:** graphene, resonators, suspended graphene, nonlinear resonance, NEMS

## Abstract

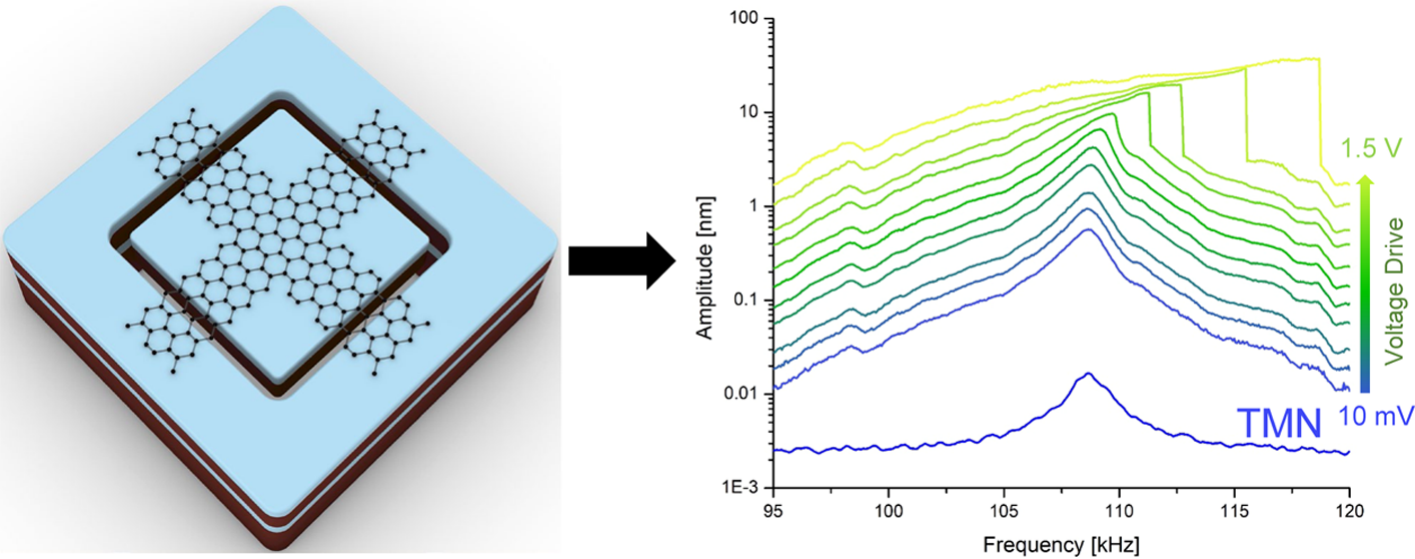

The unique mechanical and electrical properties of graphene make it an exciting material for nanoelectromechanical systems (NEMS). NEMS resonators with graphene springs facilitate studies of graphene’s fundamental material characteristics and thus enable innovative device concepts for applications such as sensors. Here, we demonstrate resonant transducers with ribbon-springs made of double-layer graphene and proof masses made of silicon and study their nonlinear mechanics at resonance both in air and in vacuum by laser Doppler vibrometry. Surprisingly, we observe spring-stiffening and spring-softening at resonance, depending on the graphene spring designs. The measured quality factors of the resonators in a vacuum are between 150 and 350. These results pave the way for a class of ultraminiaturized nanomechanical sensors such as accelerometers by contributing to the understanding of the dynamics of transducers based on graphene ribbons with an attached proof mass.

## Introduction

The ultrathin membrane thickness and excellent electrical and mechanical properties of graphene, including its Young’s modulus of up to 1 TPa,^1^ a stretchability of up to 20%,^2^ and the room-temperature electron mobility of up to 2.5 × 10^5^ cm^2^/V s,^3^ make it attractive for use in nanoelectromechanical system (NEMS) transducers, enabling the realization of small devices with the potential for fast response time, high responsivity, and wide response range. The earliest studied graphene NEMS devices were resonators that consisted of double-sided clamped single-layer graphene ribbons suspended over trenches in a SiO_2_ layer, where their mechanical properties such as fundamental resonance frequencies and quality factors were characterized at room temperature.^4^ Subsequently, different types of resonant structures^5−30^ based on suspended graphene without an attached mass were studied for the basic properties of graphene^7−9,11−13,15,17−20,23,24,26−28^ and for device applications such as ultrasensitive detection of gases,^16^ temperature,^10^ pressure,^29^ mass,^15^ vibrations,^5,31^ and for applications in fire warning^32^ and infrared spectroscopy.^14^

The resonance frequency of graphene resonators was theoretically and experimentally demonstrated to be influenced by a change in the tension of the suspended graphene that can be caused, for example, by applied electrostatic voltages,^33−36^ temperature,^10,37^ mass,^37,38^ thermal shrinkage of SU-8 resist anchors,^39^ nanoindentation forces,^40^ and external accelerations.^41,42^ Furthermore, graphene was used to study various types of nonlinear dynamic effects, such as mode-coupling, and parametric and internal resonances.^43^ As the dimensions of graphene NEMS structures shrink, their mechanical nonlinearity is reached at smaller displacements, resulting in a decreased dynamic range of NEMS devices.^15^ In contrast to suspended resonant graphene structures without an attached proof mass, there are fewer studies on resonant graphene structures with an attached proof mass. The existing studies investigated the resonance characteristics of suspended doubly clamped graphene ribbons or fully clamped graphene membranes with attached proof masses targeted at NEMS accelerometer applications.^44−47^ However, more complex graphene device designs for vibration sensing, such as a proof mass attached to four graphene ribbons (e.g., parallel-type or cross-type), have not yet been experimentally explored.

Here, we report three types of resonant NEMS structures utilizing different configurations of multiple double-layer graphene ribbons with an attached silicon (Si) proof mass. We used a laser Doppler vibrometer (LDV) to measure and analyze the resonance frequency, quality factor (*Q*), stiffness, and nonlinear resonance response of these devices. We observed unusual softening nonlinear behaviors (spring-softening) of the devices consisting of two graphene ribbons with an attached proof mass compared to the hardening nonlinear behaviors (spring-stiffening) of the devices consisting of four graphene ribbons with an attached proof mass.

## Results and Discussion

**Figure 1:**
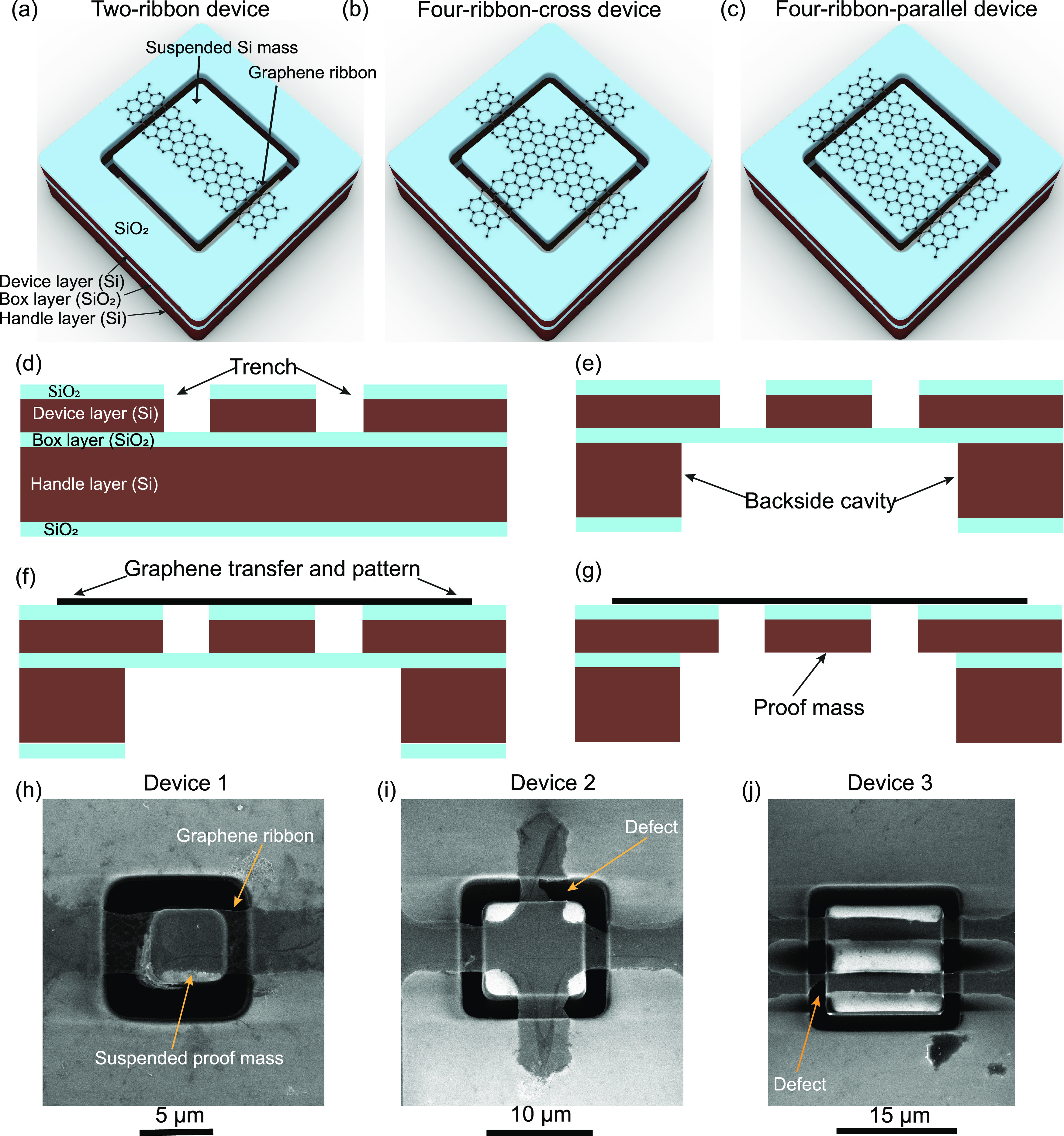
Three types of resonators based on different graphene ribbon configurations with attached proof masses. (a–c) 3D illustration of the three device designs: two-ribbon device (a); four-ribbon-cross device (b); four-ribbon-parallel device (c). (d–g) Schematic of device fabrication: trenches were etched into the oxidized Si device layer of an SOI wafer to form the Si proof masses (d); the handle layer of the SOI wafer was etched by DRIE in the areas below the proof masses (e); the double-layer graphene was transferred onto the prefabricated SOI substrate and patterned into different ribbon configurations by optical lithography and O_2_ plasma etching (f); the BOX layer of the SOI substrate was etched by RIE followed by vapor HF to release the proof masses (g). (h–j) Top-view SEM images of the three types of fabricated graphene ribbon devices: two-ribbon device (device 1) (h); four-ribbon-cross device (device 2) (i); and four-ribbon-parallel device (device 3) (j).

To systematically study the resonant properties of suspended graphene ribbons with an attached proof mass, we fabricated three device variations with different graphene ribbon configurations and dimensions of the proof mass, all consisting of suspended double-layer graphene ribbons with a Si proof mass attached at the center. The different device designs consist of (1) two graphene ribbons with an attached proof mass (Figure 1a) (two-ribbon device); (2) four crossed graphene ribbons with an attached proof mass (Figure 1b) (four-ribbon-cross device); and (3) four parallel graphene ribbons with an attached proof mass (Figure 1c) (four-ribbon-parallel device). We fabricated the devices on a thermally oxidized silicon-on-insulator (SOI) wafer. To define the Si proof masses, we first patterned a hard mask into the top SiO_2_ layer of the Si device layer by reactive ion etching (RIE) and then etched trenches into the layer by deep reactive ion etching (DRIE) (Figure 1d). At this stage, we etched cavities into the backside of the 400 μm thick Si handle layer of the SOI wafer by RIE and DRIE, thereby suspending the Si proof masses resting on the BOX layer on the front (Figure 1e). To integrate the double-layer chemical vapor deposited (CVD) graphene from the donor substrate (copper sheet) onto the surface of the prepared SOI wafer, we used PMMA-based wet transfer.^46−48^ Therefore, we did two sequential transfers to vertically stack two single layers of graphene (Graphenea, Spain) on top of each other. Next, we used optical lithography and low-power O_2_ plasma etching to pattern the graphene into the desired ribbon shapes (Figure 1f). Finally, we suspended the proof mass on the graphene ribbons by etching the exposed sections of the BOX layer (2 μm thick SiO_2_) away by dry plasma etching followed by vapor hydrogen fluoride (HF) etching (Figure 1g). Details of the device fabrication can be found in our previous reports.^46−48^ The shapes of the proof masses in all device designs were quadratic, and the thickness of the masses was 16.4 μm in all cases (a 15 μm thick Si layer and a 1.4 μm thick SiO_2_ layer at the interface to the graphene ribbons). SEM images of the three device configurations are shown in Figure 1h (device 1: two-ribbon device), Figure 1i (device 2: four-ribbon-cross device), and Figure 1j (device 3: four-ribbon-parallel device). Devices 1–3 have the same single ribbon length that is defined by the trench width (2 μm) but different ribbon widths (4 μm for device 1, 5 μm for device 2, and 3 μm for device 3) and different proof mass dimensions (5 μm × 5 μm × 16.4 μm for device 1, 10 μm × 10 μm × 16.4 μm for device 2, and 15 μm × 15 μm × 16.4 μm for device 3).

To measure the frequency response of the spring-mass systems of our graphene devices at room temperature in both air (atmospheric pressure) and vacuum, we used a laser Doppler vibrometer (Polytec OFV-5000 and OFV-551) while driving the graphene devices with a piezoshaker that converts an input signal at different driving voltages into vibrations on the *z*-axis. For all measured devices, we applied the same driving voltage amplitudes (root-mean-square (RMS) values) between 10 mV and 1.5 V. All spectra were collected with upward frequency sweeps. For reference, we also measured the thermomechanical noise (TMN) spectra of the devices in both air (atmospheric pressure) and a vacuum. We then fitted the spectra to a Lorentzian model to estimate the resonance frequencies and quality factors.

**Figure 2:**
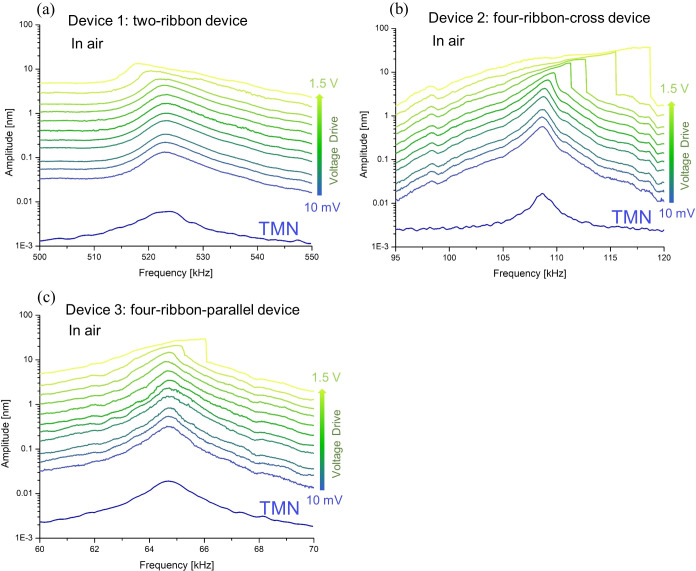
Measured frequency response of devices 1–3 with the vibration amplitude for increasing driving voltages of the piezoshaker and thermomechanical noise (TMN) measurements in air (a–c). The applied driving voltages (between 10 mV and 1.5 V) of the piezoshaker for devices 1–3 are identical.

A set of frequency response curves of the fundamental modes of device 1 (two-ribbon device), device 2 (four-ribbon-cross device), and device 3 (four-ribbon-parallel device) at different driving voltages in air are shown in Figure 2a–c, respectively. In all devices, the vibration amplitude at resonance increased with increasing driving voltage. For low driving voltages (<75 mV), the vibration amplitudes were low (<0.5 nm) and the frequency response was linear. Further, as expected, the resonance frequency and *Q* of each device measured from their thermomechanical noise spectra were similar to those measured using the laser Doppler vibrometer at low piezoshaker driving voltages (Figure 2 and Table 1). Based on the resonance frequencies of devices 1–3, the built-in stress in the graphene ribbons can be estimated to be in the range of 500 MPa up to 3.7 GPa and the corresponding built-in tension can be estimated to be in the range of 1.4–10 μN. The stress for device 1 is around 5–7 times higher than that for devices 2 and 3. The built-in tension in the suspended graphene ribbons can be impacted by the design of devices, the process of transferring graphene, the final graphene substrate surface, and the graphene source material.^46^ A part of the build-in tension in the suspended graphene ribbon is mainly determined by the geometry at the graphene anchor points and by the strength of the van der Waals interactions between the graphene and the SiO_2_ surface at the microscopically rounded edges and sidewalls of the etched trenches.^46^

**Table 1:** Resonance Frequency (*f*) and Quality Factor (*Q*) of Devices 1–3 Measured in Air and Vacuum

		device 1	device 2	device 3
in air	*f* (kHz) at 10 mV	523	109	65
	*Q* at 10 mV	78	78	73
in vacuum	*f* (kHz) at 10 mV	527	131	72
	*Q* at 10 mV	155	241	182

At high driving voltages of the piezoshaker (>120 mV), the frequency response curves of devices 1–3 start to show nonlinear behavior and the resonance peaks become asymmetric, as expected.^49^ Interestingly, device 1 (two-ribbon) showed a softening behavior (Figure 2a), while device 2 (four-ribbon-cross) and device 3 (four-ribbon-parallel) showed a hardening behavior (Figure 2b,c). That is, the resonance frequency of device 1 decreased with an increase of the driving voltages, while the resonance frequency of devices 2 and 3 increased with an increase of the driving voltages. Animated GIFs taken using a digital holographic microscope of the motion of devices 1–3 in air at a driving voltage of 1 V are provided in Videos S1–S3, respectively.

**Figure 3:**
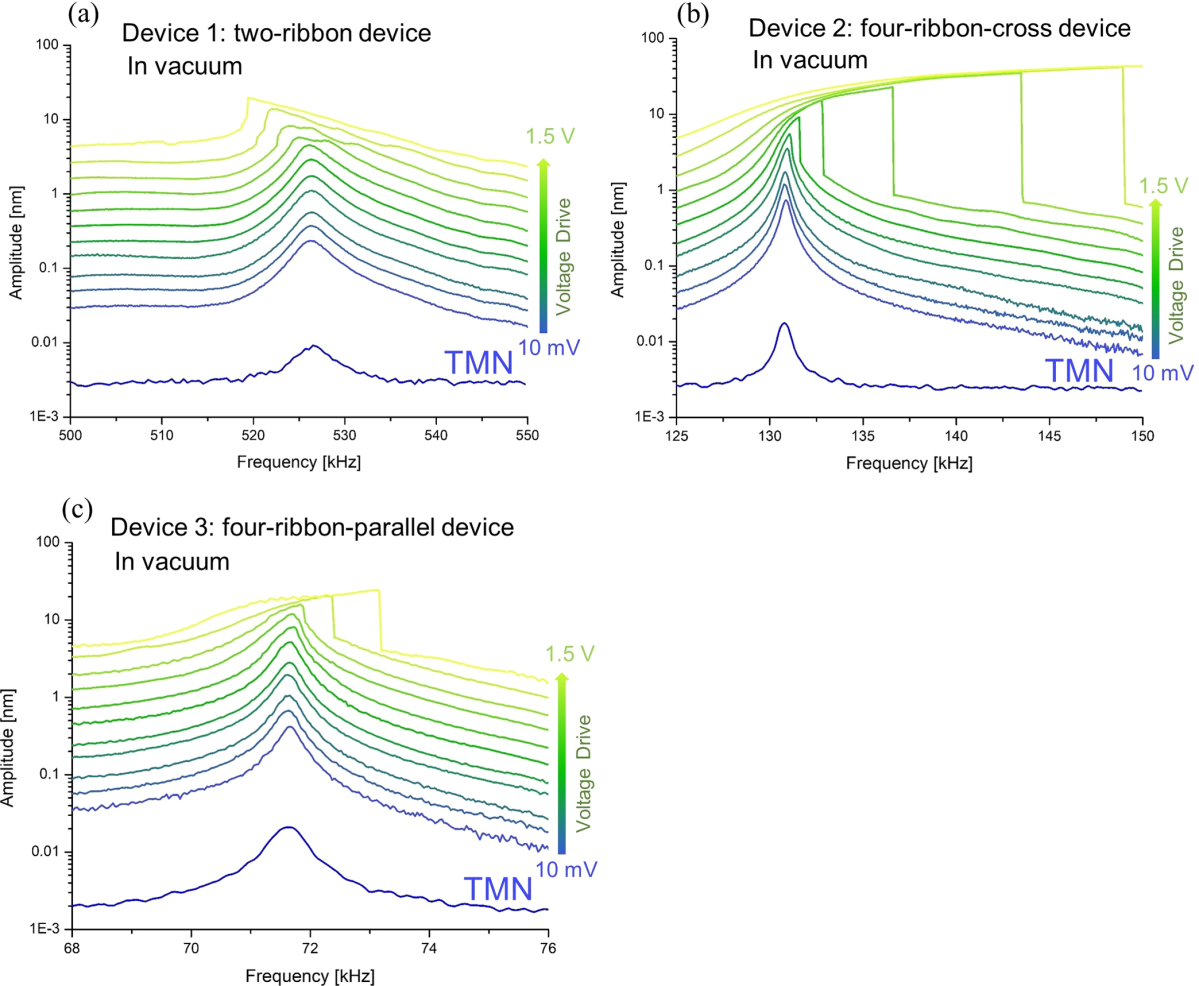
Measured frequency response of devices 1–3 with the vibration amplitude for increasing driving voltages of the piezoshaker and thermomechanical noise (TMN) measurements in a vacuum (a–c). The applied driving voltages (between 10 mV and 1.5 V) of the piezoshaker for devices are identical.

To explore if similar softening and hardening behaviors of devices 1–3 occur in a vacuum, we measured a set of frequency response curves of the fundamental mode of devices 1–3 for a range of driving voltages of the piezoshaker in a vacuum (Figure 3a–c). At low driving voltages, e.g., 10 mV, the extracted resonance frequencies and *Q* of devices 1–3 in vacuum were 527, 131, 72 kHz and 155, 241, 182, respectively (Table 1). Thus, the extracted *Q* in vacuum was 2–3 times higher than in air, indicating that a significant part of the losses in the resonators are due to either gas damping or surface absorbates that desorb when in vacuum. As is the case in air, we observed the softening behavior in device 1 at high driving voltages in vacuum, while in devices 2 and 3, we observed the hardening behaviors, indicating that this feature is consistent in both air and vacuum operation of the resonators.

To evaluate the reproducibility of the softening behavior in graphene devices containing a two-ribbon configuration, we measured the frequency response of another two-ribbon device (device 4: single ribbon length of 2 μm, ribbon width of 8 μm, proof mass dimensions of 10 μm × 10 μm × 16.4 μm) in both air and vacuum by laser Doppler vibrometry under identical conditions as we measured devices 1–3 (Figure S1). At high driving voltages, we observed again the softening behavior in both air and vacuum, consistent with the characteristics observed in device 1.

Observing a nonlinear response in our resonators is not surprising. Any clamped–clamped structure is going to show built-in tension when the amplitude of the motion increases.^49^ This expected nonlinear behavior is what we call geometric nonlinearity associated with the mode shape and motion, and it is always hardening, meaning that the Duffing coefficient α > 0. For the case of graphene, or other 2D resonators, it has been reported repeatedly in the past.^15,25,43,50,51^ For our type of devices (with a Si mass in the center) but without ribbons, we have also shown this in the past.^31^ What makes our two-ribbon devices special is that their nonlinear behavior is softening, meaning α < 0. This is somewhat unexpected.^15,43,50,51^

Indeed, the spring hardening of device 2 (four-ribbon cross device) and device 3 (four-ribbon parallel device) can be ascribed to the stress-built-up in the ribbons during the mass motion. This is the usual behavior of any clamped–clamped resonator structure with large aspect ratios (*L*/*t*), including resonators made of graphene, silicon, and other materials.^49^ The increased driving voltage results in the observed increase in the deflection and stiffness of the graphene ribbons and the tilting to the right of the “quasi-Lorentzian” response for high drives. This geometric effect yields a critical amplitude of around , where *L* is the ribbon length, *Q* is the quality factor of the device, σ_0_ is the built-in stress, and *E* is Young’s modulus of the graphene membrane. Evaluating this expression using the extracted value for the stress, it yields around 7 nm in the case of devices 2 and 3, while it yields values above 30 nm for devices 1 and 4. This explains why the stiffening behavior stemming from geometric nonlinearity is visible in those two devices and not the others.

What is more interesting is the analysis of the devices with 2 ribbons, which shows unusual softening nonlinearity. Typically, one can ascribe such a nonlinear behavior to some transduction effect, for example, capacitive driving under certain conditions.^25,43,52^ In our case, since all of our devices under test were driven by a piezoshaker and detected in the same way, this is not a possible explanation. However, we could find an explanation in the material nonlinearity for graphene, which has been reported to be reached for strain values of around 5%.^53^ For device 1, the built-in strain is already around 1%, which means that it is on the same order of magnitude with the material nonlinearity limit. This effect is typically neglected because there are other nonlinearities that arise before material softening. However, in this case, it could be a possibility. Another possible reason could be the partial delamination of the graphene ribbons from the SiO_2_ surface of the edges of the proof mass or the trench edges in devices 1 and 4. Since only two ribbons are present in devices 1 and 4, the effective force per unit length that graphene observes is larger than in devices 2 and 3, where four ribbons are present. Although there exist strong van der Waals interactions between the graphene and the SiO_2_ surface,^46,54^ the calculated force per ribbon is larger in the devices exhibiting softening nonlinearities (Table S1 and related text in the Supporting Information). This possible delamination would make the graphene ribbons of devices 1 and 4 longer during vibration and their resonance frequencies would decrease consequently.

In addition, this possible delamination of the graphene ribbons of the two-ribbon devices away from the SiO_2_ surface of the edges of the Si mass or the trench edges at nonlinear resonances would probably result in the decrease of built-in stress of graphene ribbons to some extent and likely contribute to the Duffing softening behavior that we observed in the two-ribbon devices (devices 1 and 4). The possible lamination of the graphene ribbons of two-ribbon devices to the SiO_2_ surface of the edges of the Si mass or the trench edges would probably result in the recovery of the built-in stress. The competition between elastic mechanisms (stretching of graphene ribbons)^25,37,43,50,52^ and the decrease of built-in stress in graphene ribbons could result in the observed hardening or softening Duffing behavior.

The different types of graphene ribbons with attached proof mass fabricated can be potentially used as NEMS transducers for their applications in ultra-small NEMS accelerometers^46,48,55^ and vibration sensors.^31^ Compared to the two-ribbon devices that were explored previously,^46^ the four-ribbon devices would potentially provide higher device manufacturing yields, improved mechanical stability, and longer lifetime.

## Conclusions

In conclusion, we have reported three types of devices with different graphene ribbon configurations with attached proof masses for use as NEMS resonators to study nonlinear resonance behavior, including two-ribbon devices, four-ribbon cross-devices, and four-ribbon-parallel devices. We measured, compared, and analyzed the resonance frequencies, quality factors, spring constant, and nonlinear resonance behavior of all three device types in air and vacuum. We found that our two-ribbon devices showed unexpected softening behavior compared with the hardening behavior of the four-ribbon devices. The study of graphene NEMS devices with different types of graphene ribbon configurations and attached proof masses will lead to a better understanding of the dynamics of graphene and other 2D material membranes and their applications in NEMS resonators and accelerometers.

## Supporting Information Available

Videos S1–S3: animated GIF taken using a digital holographic microscope of the motion of devices 1–3 (ZIP)
Captions to Videos S1–S3 (PDF)
Figure S1: measured frequency response of device 4; Table S1: analysis of force per graphene ribbon of devices 1–4 (PDF)
