# Supplementary figures and images for: Resonant Transducers Consisting of Graphene Ribbons with Attached Proof Masses for NEMS Sensors
Source: ACS Appl Nano Mater. 2023 Dec 1;7(1):102–9. doi: 10.1021/acsanm.3c03642 (PMC10788872; doi:10.1021/acsanm.3c03642)

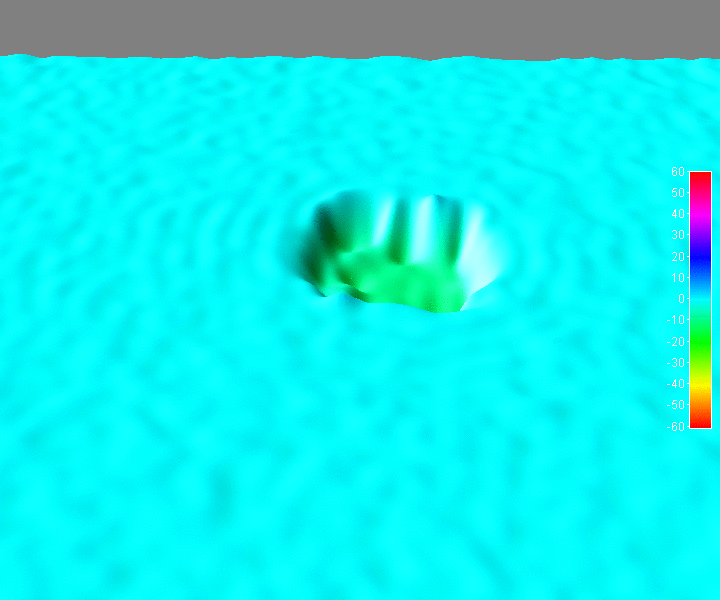

Supplement: Supplementary file 1 — an3c03642_si_001.zip [file an3c03642_si_001.zip › Supplementary Video 1.gif]

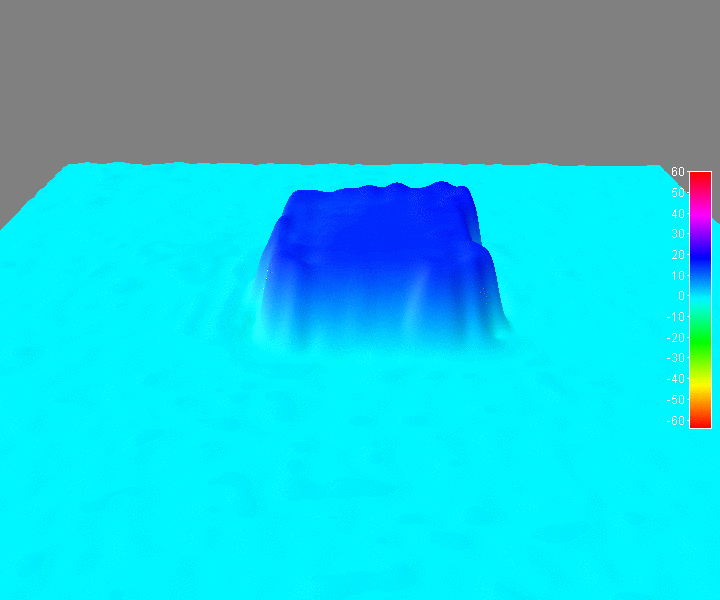

Supplement: Supplementary file 1 — an3c03642_si_001.zip [file an3c03642_si_001.zip › Supplementary Video 2.gif]

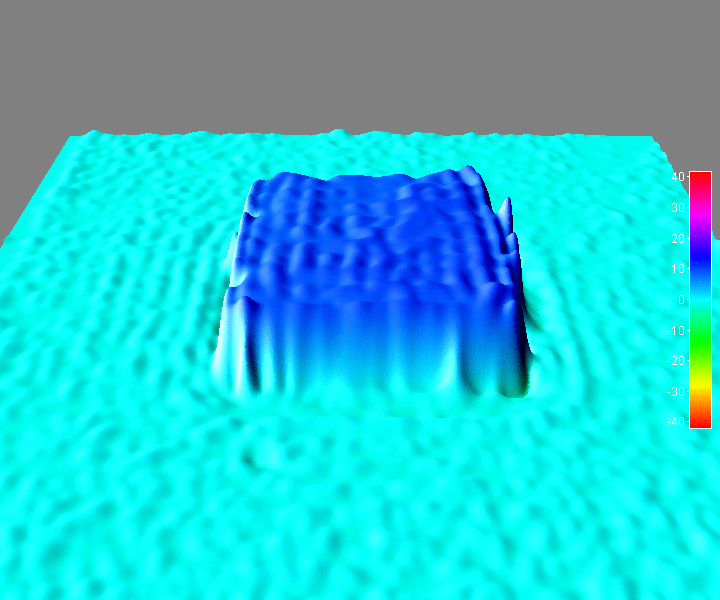

Supplement: Supplementary file 1 — an3c03642_si_001.zip [file an3c03642_si_001.zip › Supplementary Video 3.gif]
