# Supplementary material for: Resonant Transducers Consisting of Graphene Ribbons with Attached Proof Masses for NEMS Sensors
Source: ACS Appl Nano Mater. 2023 Dec 1;7(1):102–9. doi: 10.1021/acsanm.3c03642 (PMC10788872; doi:10.1021/acsanm.3c03642)
Supplement: Supplementary file 2 — an3c03642_si_002.pdf [file an3c03642_si_002.pdf]

Supplementary Video 1: Animated GIF taken using a Digital Holographic Microscope of the motion of device 1 (two-ribbon device) in air at a driving voltage of 1 V, with the resonance frequency of 568 kHz. The increase of resonance frequency of device 1 from about 520 kHz (Fig. 2a) to 568 kHz in air at the driving voltage of 1V can be ascribed to the possible "cleaning" of the graphene ribbons' surface after vacuum or the possible warming up effect of laser used for the measurements. Device 1 has the single ribbon length of 2 μm, ribbon width of 4 μm, and proof mass dimensions (5 μm × 5 μm × 16.4 μm).

Supplementary Video 2: Animated GIF taken using a Digital Holographic Microscope of the motion of device 2 (four-ribbon-cross device) in air at a driving voltage of 1 V, with the resonance frequency of 118.8 kHz. Device 2 has the single ribbon length of 2 μm, ribbon width of 5 μm, and proof mass dimensions (10 μm × 10 μm × 16.4 μm).

Supplementary Video 3: Animated GIF taken using a Digital Holographic Microscope of the motion of device 3 (four-ribbon-parallel device) in air at a driving voltage of 1 V, with the resonance frequency of 72.7 kHz. Device 3 has the single ribbon length of 2 μm, ribbon width of 3 μm, and proof mass dimensions (15 μm × 15 μm × 16.4 μm).
