# Supplementary material for: Resonant Transducers Consisting of Graphene Ribbons with Attached Proof Masses for NEMS Sensors
Source: ACS Appl Nano Mater. 2023 Dec 1;7(1):102–9. doi: 10.1021/acsanm.3c03642 (PMC10788872; doi:10.1021/acsanm.3c03642)
Supplement: Supplementary file 3 — an3c03642_si_003.pdf [file an3c03642_si_003.pdf]

Supporting Information

Resonant Transducers Consisting of Graphene Ribbons with Attached Proof Masses for Vibration Sensing

Xuge Fan[1,2,*], Daniel Moreno-Garcia[3], Jie Ding[4], Kristinn B. Gylfason[2], Luis Guillermo Villanueva[3,*], and Frank Niklaus[2,*]

[1]Advanced Research Institute of Multidisciplinary Sciences, Beijing Institute of Technology, 100081 Beijing, China.

[2]Division of Micro and Nanosystems, School of Electrical Engineering and Computer Science, KTH Royal Institute of Technology, SE-10044 Stockholm, Sweden.

[3]Advanced NEMS Group, École Polytechnique Fédérale de Lausanne (EPFL), 1015 Lausanne, Switzerland.

[4]School of Integrated Circuits and Electronics, Beijing Institute of Technology, 100081 Beijing, China.

*Email: frank@kth.se, guillermo.villanueva@epfl.ch, xgfan@bit.edu.cn

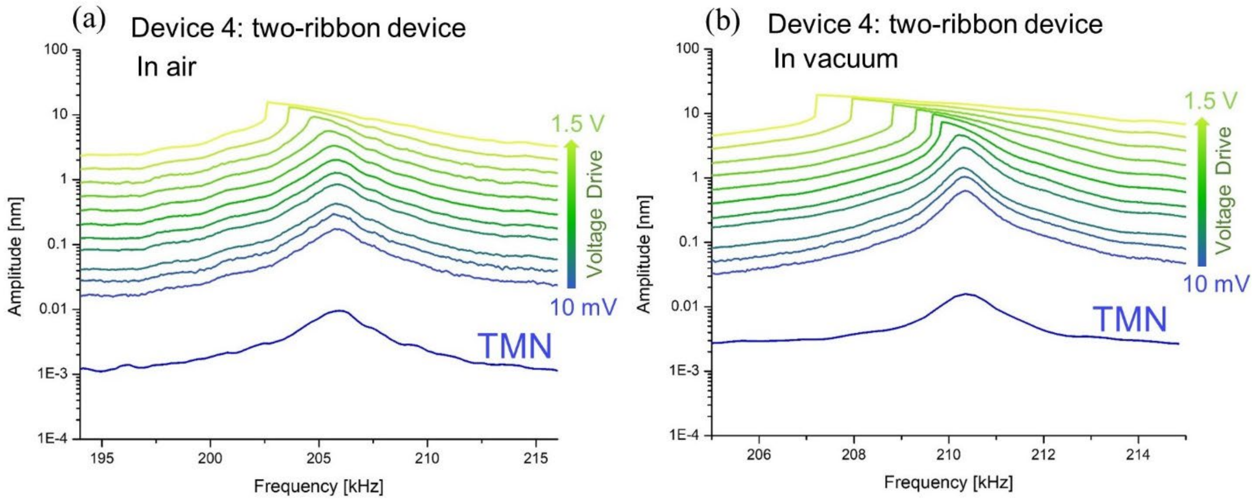

Figure S1. Measured frequency response of device 4 with the vibration amplitude for increasing driving voltages of the piezoshaker and the thermomechanical noise (TMN) measurements in air (a) and vacuum (b). The applied driving voltages of the piezoshaker are between 10 mV and 1.5 V. At low driving voltages, the resonance frequency of device 4 in air and vacuum was about 206 kHz and 210 kHz, respectively. The extracted Q of device 4 in vacuum was 332, about 3 times higher than the Q of 102 in air. At high driving voltages, we observed again the unusual softening nonlinear behaviour in both air and vacuum, consistent with the characteristics observed in device 1. Again, the softening nonlinear behaviour of device 4 in vacuum also was much stronger than in air.

Table S1. Resonance frequency (f), spring constant (K) of ribbon-mass system, maximum displacement of proof mass, and forces acting on graphene ribbons of devices 1-4 measured in air and vacuum.

		Device 1	Device 2	Device 3	Device 4
Weight of mass (nN)		0.01	0.04	0.09	0.04
In air	*f* (kHz)	523	109	65	206
	K (N/m)	10	1.7	1.4	6.2
	Deflection (nm) at 1.5V	15.3	37.1	29.4	17.2
	Force (nN) (All ribbons)	157.8	65.3	42.2	110.1
	Force (nN) (Each ribbon)	78.9	16.3	10.6	55.1
In vacuum	*f* (kHz)	527	131	72	210
	K (N/m)	10	2.5	1.7	6.2
	Deflection (nm) at 1.5 V	19.2	43.0	27.0	22.5
	Force (nN) (All ribbons)	200.3	111.3	47.5	149.7
	Force (nN) (Each ribbon)	100.2	27.8	11.9	74.9

For all measured devices, we applied the same driving voltage amplitudes (Root Mean Square (RMS) values) between 10 mV and 1.5 V (10 mV, 16.5 mV, 25 mV, 50 mV, 75 mV, 122.6 mV, 202 mV, 334 mV, 551 mV, 910.5 mV and 1.5 V). As shown in **Table S1**, the resonance frequencies of devices 1-4 are approximately 523 kHz, 109 kHz, 65 kHz and 206 kHz in air and 527 kHz, 131

kHz, 72 kHz and 210 kHz in vacuum, respectively. Therefore, the effective spring constants of the ribbon-mass systems of devices 1-4 are on the order of 10 N/m, 1.7 N/m, 1.4 N/m and 6.2 N/m in air and 10 N/m, 2.5 N/m, 1.7 N/m and 6.2 N/m in vacuum, respectively (**Table S1**). The maximum displacements of the suspended proof masses of devices 1-4 at nonlinear resonances at a driving voltage of 1.5 V are approximately 15.3 nm, 37.1 nm, 29.4 nm and 17.2 nm in air, and 19.2 nm, 43.0 nm, 27.0 nm and 22.5 nm in vacuum (**Table S1**). And according to Hooke's law $F = Kx$, the forces acting on the graphene ribbons of devices 1-4 at nonlinear resonances in air and in vacuum are on the order of tens to hundreds of nN (**Table S1**). For reference, the forces acting on proof masses of devices 1-4 (5 μm × 5 μm × 16.4 μm) due to gravity are of the order of 0.01 nN, 0.04 nN, 0.09 nN and 0.04 nN, all of which are three to four orders of magnitude smaller than the forces acting on the graphene ribbons of devices 1-4 at the nonlinear resonances. The force acting on each single graphene ribbon of devices 1 and 4 at nonlinear resonances at a driving voltage of 1.5 V are about three to seven times higher than that acting on each single graphene ribbon of devices 2 and 3. Therefore, the possible delamination of the graphene ribbons of devices 1 and 4 away from the SiO_2 surface of the edges of the Si mass or the trench edges at nonlinear resonances more easily occurs than in devices 2 and 3 at a given driving voltage (e.g. 1.5 V). This possible delamination would result in the increase of the length of graphene ribbons of devices 1 and 4, which could contribute to the softening behaviour we observed.
